# Impact of Frontier Development of Alveolar Bone Grafting on Orthodontic Tooth Movement

**DOI:** 10.3389/fbioe.2022.869191

**Published:** 2022-06-30

**Authors:** Yilan Miao, Yu-Cheng Chang, Nipul Tanna, Nicolette Almer, Chun-Hsi Chung, Min Zou, Zhong Zheng, Chenshuang Li

**Affiliations:** ^1^ School of Dental Medicine, University of Pennsylvania, Philadelphia, PA, United States; ^2^ Department of Periodontics, School of Dental Medicine, University of Pennsylvania, Philadelphia, PA, United States; ^3^ Department of Orthodontics, School of Dental Medicine, University of Pennsylvania, Philadelphia, PA, United States; ^4^ Key Laboratory of Shannxi Province for Craniofacial Precision Medicine Research, College of Stomatology, Xi’an Jiaotong University, Xi’an, China; ^5^ Clinical Research Center of Shannxi Province for Dental and Maxillofacial Diseases, College of Stomatology, Xi’an Jiaotong University, Xi’an, China; ^6^ Department of Orthodontics, College of Stomatology, Xi’an Jiaotong University, Xi’an, China; ^7^ David Geffen School of Medicine, University of California, Los Angeles, Los Angeles, CA, United States; ^8^ School of Dentistry, University of California, Los Angeles, Los Angeles, CA, United States

**Keywords:** orthodontic tooth movement, alveolar bone graft, novel material, BMP-2, platelet-rich fibrin (PRF), bioactive glass, stem cell

## Abstract

Sufficient alveolar bone is a safeguard for achieving desired outcomes in orthodontic treatment. Moving a tooth into an alveolar bony defect may result in a periodontal defect or worse–tooth loss. Therefore, when facing a pathologic situation such as periodontal bone loss, alveolar clefts, long-term tooth loss, trauma, and thin phenotype, bone grafting is often necessary to augment bone for orthodontic treatment purposes. Currently, diverse bone grafts are used in clinical practice, but no single grafting material shows absolutely superior results over the others. All available materials demonstrate pros and cons, most notably donor morbidity and adverse effects on orthodontic treatment. Here, we review newly developed graft materials that are still in the pre-clinical stage, as well as new combinations of existing materials, by highlighting their effects on alveolar bone regeneration and orthodontic tooth movement. In addition, novel manufacturing techniques, such as bioprinting, will be discussed. This mini-review article will provide state-of-the-art information to assist clinicians in selecting grafting material(s) that enhance alveolar bone augmentation while avoiding unfavorable side effects during orthodontic treatment.

## Introduction

To avoid fenestrations or dehiscences during orthodontic tooth movement, it is critical for alveolar bone to possess adequate contour, thickness, and quality (Atwood and Coy, 1971; Abrams et al., 1987; Seifi and Ghoraishian, 2012). Orthodontically moving teeth into a region with reduced alveolar bone can worsen the periodontal status, slow down tooth movement, and cause root resorption or even tooth loss (Reichert et al., 2010). Clinical scenarios such as severe periodontitis, congenital alveolar clefts, long-term tooth loss, and trauma can induce alveolar bone loss (McAllister and Haghighat, 2007). Thus, augmentation of insufficient bone volume is often indicated prior to the initiation of orthodontic treatment.

In addition, patients with a thin phenotype have narrow alveolar bone support, which significantly limits the range of orthodontic tooth movement. To address this issue, the periodontally accelerated osteogenic orthodontics (PAOO) technique has been developed to broaden the biological range of orthodontic treatment by adding bone grafting material to the alveolar cortical surface (Wilcko et al., 2008). Pre-orthodontic bone grafting can also promote easier and less detrimental tooth movement through primary woven bone (Diedrich, 1996). Ideally, bone graft materials for orthodontic treatment should protect the teeth from complications and enhance the alveolar bone phenotype.

Based on where bone grafts are sourced, they may be categorized as autografts, allografts, xenografts, or synthetics. Autografts prevail amongst these categories in the maxillofacial region and are the current gold standard as they [1] consist of an abundance of spongy bone that is close to the alveolar bone structure, [2] display osteoconductive and osteoinductive potential (Boyne and Sands, 1972; Enemark et al., 1987; Ozaki and Buchman, 1998), and [3] promote periodontal regeneration (Ivanovic et al., 2014) without significantly unfavorable sequelae when teeth are orthodontically moved into grafted areas (Lu et al., 2021). However, the drawbacks of autografts are substantial, including but not limited to inadequate availability, expensive cost, mismatched size, and inevitable additional surgery for autograft harvest (Sharif et al., 2016). These limitations lend support to the use of substitute graft materials.

Allografts, such as decalcified freeze-dried bone allogeneic grafts (DFDBA) and freeze-dried bone allogeneic grafts (FDBA), are orthodontic-friendly (Lu et al., 2021); however, their osteoinductive potency is not conclusive (Schwartz et al., 1998). Xenografts, such as Bio-Oss® and Gen-Tech®, are the most common alveolar grafting materials for clinical use. They are successful when used for alveolar bone augmentation (da Silva et al., 2020), but can severely impair orthodontic treatment and cause substantial root resorption when teeth are moved into the grafted region (Lu et al., 2021). Although synthetic bone grafts, such as NanoBone® and BoneCeramic®, also promote bone augmentation, major adverse effects (namely root resorption and gingival invagination) make them an unfavorable choice for pre-orthodontic alveolar bone grafting (Lu et al., 2021). Therefore, there is an emerging need for new grafting materials to be not only osteoinductive and osteoconductive but also supportive of highly active bone metabolism during orthodontic tooth movement without adverse effects.

In this review, we highlight recent research advances in novel alveolar graft materials, as well as new combinations of previously developed materials, with a focus on orthodontic applications supported by pre-clinical and clinical evidence (Table 1).

**TABLE 1:** The alveolar bone regeneration efficiency and the orthodontic impactions of the alveolar bone grafting materials.

Materials	References	Combinatory Materials	Type of Study	Alveolar Bone Regeneration Efficiency		Side Effects			Impact on Orthodontics	
				Volume	Cellular Activity	Inflammation	Pain	Graft Failure	Tooth Movement Rate	Adverse Effect
BMP2	Kawamoto et al. (2002)	poly [_D,L_-(lactide-*co*-glycolide)]/gelatin sponge complex	Animal study (dog)	Significantly greater regenerated bone than spongiosa autograft	More osteoinductive activity associated with rhBMP2	N/A	N/A	N/A	Both rhBMP2 and spongiosa groups showed similar responses to orthodontic force as normal alveolar bone	Root resorption on pressure side with rhBMP2
	Hammoudeh et al. (2017)	DBM scaffold	Clinical study (secondary alveolar cleft repair)	Comparable bone regrowth and density as autologous iliac crest bone graft	N/A	Self-limited facial swelling, minor wound dehiscence	Improved without intervention	No increase in serious adverse events compared to iliac bone graft	Similar spontaneous canine eruption rate was observed among rhBMP2 and iliac crest bone groups	N/A
	Chandra et al. (2019)	N/A	Clinical study (PAOO)	A highly significant increase in bone density compared to conventional corticotomy procedure	BMP-2 stimulates recruitment and differentiation of osteoclasts	No significant difference on wound healing	No significant difference on pain scores	N/A	Reduced orthodontic treatment time	N/A
	Jiang et al. (2020)	BMP2-functionalized BioCaP granules	Animal study (dogs)	Compared to bovine xenograft: 1.25-fold enhanced bone formation, 1.42-fold more graft resorption, 1.36-fold higher bone density	BMP mediated osteogenesis-angiogenesis coupling	Reduced inflammation compared to bovine xenograft	Not observed	N/A	Slightly reduced orthodontic tooth movement rate but statistically not significant compared to bovine xenograft	Less root resorption and reduced periodontal probing depth compared to bovine xenograft
β-TCP	de Ruiter et al. (2011)	N/A	Animal study (goats)	More bone ingrowth than autografted iliac bone grafts, but the difference was not significant	No significant difference between β-TCP and iliac bone groups	No significant difference	N/A	N/A	No difference in orthodontic tooth movement between β-TCP and iliac bone	Minor degree of apical root resorption, analogous with human situation
	Klein et al. (2019)	N/A	Animal study (mice)	β-TCP and long bone allograft both induce normal bone healing, similarly to non-grafted normally healing sites	Increased osteoclast recruitment induced by β-TCP at the early stages of healing compared to allograft using long bones	No adverse inflammatory response	Not observed	Not observed	β-TCP and allograft both slowed orthodontic movement compared to control without grafting; no difference in orthodontic movement between β-TCP and allografts	N/A
Bioactive glasses	El Shazley et al. (2016)	N/A	Clinical study (extraction socket preservation)	TAMP grafted sockets healed with vertical trabeculae and large vascularized marrow spaces; better preservation of socket contour	TAMP scaffolds enhanced the recruitment of stem cells from grafted sockets	N/A	N/A	Not observed	N/A	N/A
	Shoreibah et al. (2012)	N/A	Clinical study (PAOO)	Significantly higher bone density was observed with bioactive glasses compared to the control group without grafting	Bioactive glass particles attract osteoprogenitor cells and osteoblasts	N/A	N/A	N/A	Significant reduction in total treatment time compared to the control group without grafting	No statistical difference on root resorption; absence of any significant apical root resorption
	Bahammam, (2016)	N/A	Clinical study (PAOO)	Lower bone density than bovine xenograft but not statistically significant. Both bioactive glass and bovine xenograft showed significantly greater density than the control group without grafting	Bioactive glass has homeostatic properties and demonstrated both osteoprotection and osteoconduction	Not observed	Not observed	Not observed	No difference was observed among bioactive glass, bovine xenograft, and control (no graft) groups	No significant difference in root length in all bioactive glass, bovine, and control (no graft) groups
PRF	Tehranchi et al. (2018)	N/A	Clinical study (extraction socket preservation)	Significantly higher bone density than control group without grafting	PRF contains various growth factors, cytokines, and enzymes	N/A	15% of patients reported severe post-injection pain	N/A	PRF accelerated orthodontic tooth movement, particularly in extraction cases	N/A
	Sar et al. (2019)	N/A	Animal study (rabbits)	N/A	PRF membrane alone led to an almost 3 times higher osteoblast cell count and almost 2.5 times higher blood vessel count when compared to the untreated control	Not observed	Not observed	N/A	PRF accelerated tooth movement	No orthodontic-related discomfort was observed
BM-MSCs	Tanimoto et al. (2015)	N/A	Animal study (dogs)	Radiopaque newly formed bone was observed with periodontal ligament space using MSCs, whereas the bone on carbonated hydroxyapatite control group is immature	MSCs exert new bone formation by osteogenic differentiation and induce capillary vessels	N/A	N/A	N/A	No difference in amount of tooth movement compared to carbonated hydroxyapatite for control; MSCs exhibit consistent tooth movement rate but control group did not	Not observed

rhBMP-2: recombinant human bone morphogenetic protein-2; DBM: demineralized bone matrix; PAOO: periodontally accelerated osteogenic orthodontics; β-TCP: beta tricalcium phosphate; TAMP scaffold: tailored amorphous multiporous scaffold; PRF: platelet-rich fibrin; BM-MSC: bone marrow-derived mesenchymal stromal cells; OTM: orthodontic tooth movement.

## Osteoinductive Growth Factor Bone Morphogenetic Protein 2 (BMP2)

Growth factors, cytokines, and chemokines that potentially enhance osteoblast proliferation and function as well as facilitate orthodontic tooth movement have been investigated for use as bone graft materials. For example, recombinant human BMP2 (rhBMP2), a potent osteogenic growth factor, is currently the only Food and Drug Administration (FDA)-approved osteoinductive growth factor for bone graft substitutes (James et al., 2016). In the alveolar region, animal studies show that rhBMP2 with a poly [_D,L_-(lactide-*co*-glycolide)]/gelatin sponge complex has superior osteoinductive activity compared to spongiosa from the tibia, and the newly generated bone in both groups shows a similar histological response to orthodontic force as that of normal alveolar bone (Kawamoto et al., 2002). However, root resorption was observed over the 6-months course of tooth movement when the rhBMP2-based graft was used, while no significant resorption was observed in the autograft and control groups (Kawamoto et al., 2002). Moreover, Hammoudeh’s group showed comparable bone regrowth and density values following secondary alveolar cleft repair in humans using a rhBMP2/DBM scaffold with an autologous iliac bone graft (Hammoudeh et al., 2017; Liang et al., 2017). The spontaneous canine eruption rate was similar among different grafting groups (Hammoudeh et al., 2017). In addition, applying rhBMP2 during PAOO procedures increased bone density around corticotomy sites and shortened orthodontic treatment time compared to conventional corticotomy alone (Chandra et al., 2019).

It is worth noting that although the osteoinductive activity of rhBMP2 increases with dose (El Bialy et al., 2017), high-dose rhBMP2 may not be favorable for orthodontic tooth movement. Kawamoto *et al.* found that high-dose rhBMP2 delays bone remodeling compared to low-dose rhBMP2 (Kawamoto et al., 2003). Moreover, high-dose rhBMP2 induces root resorption, while low-dose rhBMP2 causes only partial cementum resorption on the pressure side (Kawamoto et al., 2003).

To minimize the adverse effects of high-dose rhBMP2 while reducing the cost of this expensive material, rhBMP2-functionalized biomimetic calcium phosphate (BioCap) granules have been developed to achieve controlled and sustained rhBMP2 release. BioCap granules robustly enhanced bone regeneration and graft degradation over deproteinized bovine bone in an animal study (Jiang et al., 2020). In addition, due to its low immunogenicity and high angiogenic potency, BioCap graft reduces inflammation and periodontal probing depth during orthodontic treatment, while only slightly reducing the rate of orthodontic tooth movement (Jiang et al., 2020).

A synergistic effect was observed when rhBMP2 and vascular endothelial growth factor (VEGF) were used together to enhance bone generation around implant sites via an insoluble collagenous bone matrix (Schorn et al., 2017). In this combination, VEGF promotes angiogenesis and enhances osteoblastic differentiation, thereby facilitating craniofacial ossification (Duan et al., 2016), while the matrix acts as a scaffold for migrating osteoblasts. This combination product can reduce surgery time and minimize donor site morbidity while maintaining bone stability, as little resorption was observed over time (Schorn et al., 2017).

Despite its advantages, clinical complications such as significant postoperative facial swelling were observed in patients grafted with rhBMP2 (Hammoudeh et al., 2017). Along with increasing clinical use of rhBMP2 in orthopedics, a growing side-effect profile has emerged, including postoperative inflammation, ectopic bone formation, osteoclast-mediated bone resorption, and inappropriate adipogenesis (James et al., 2016). BMP2 has also been associated with osteosarcoma growth (Tian et al., 2019); this complication has cast doubt on its application after tumor resection. Safe application of rhBMP2 therefore remains an inherent issue to conquer.

## Synthetic Inorganic Materials

Unlike autografts, allografts, and xenografts, synthetic materials are free from cross-infection and disease transmission and are not associated with donor site sacrifice. However, synthetic materials, particularly inorganic ones, are often osteoconductive without any osteoinductive or osteogenic potential. β-tricalcium phosphate (β-TCP), hydroxyapatite, and bioactive glasses are the most commonly used inorganic graft materials in periodontal regeneration (Sheikh et al., 2017).

### β-tricalcium Phosphate (β-TCP)

TCPs were the first generation of calcium compounds used as bone grafts (Bohner et al., 2020). They are osteoconductive and have a similar composition to bone minerals. TCP has two crystallographic forms, α-TCP and β-TCP (Bohner et al., 2020), with the latter exhibiting good biocompatibility and osteoconductivity. As a graft material for alveolar cleft repair in animals, β-TCP promotes bone regeneration as effectively as autologous iliac crest bone (de Ruiter et al., 2011) and allograft from long bones (Klein et al., 2019). Moreover, no difference in orthodontic movement is observed between β-TCP and autograft (de Ruiter et al., 2011) or allograft (Klein et al., 2019). Since β-TCP shows no significant adverse effects on tooth movement in grafted sites, it is a promising material for further clinical investigation.

### Bioactive Glasses

First introduced as a bone graft in early 1970, biocompatible tissue-bonding bioactive glasses are another synthesized inorganic graft material that has received clinical attention. After implantation, a hydroxycarbonate apatite layer and silicon-rich gel layer form on the surface of the bioactive glass. The roles of these layers are to attach to the surrounding bone and attract osteoprogenitor cells and osteoblasts, respectively (Hench, 1991). The composition of a particular bioactive glass (i.e. a combination of silicon dioxide, calcium oxide, sodium oxide, and phosphorus pentoxide) will determine its bioactivity (Shue et al., 2012). For instance, increasing silicon dioxide, decreasing alkali, and supplementing aluminum oxide modulates the durability and water resistance of bioactive glass, thereby altering its reliability and success (Pereira et al., 1994).

Different types of bioactive glass have been tested for alveolar bone grafting and novel modifications have been developed to improve biocompatibility of the material. For example, a novel bioactive glass scaffold, tailored amorphous multiparous (TAMP), was introduced in 2016 for extraction socket preservation (El Shazley et al., 2016). Distinct from non-grafted sockets that showed corticalization after healing, the TAMP-grafted sockets healed with vertical trabeculae and large vascularized marrow spaces (El Shazley et al., 2016). Better preservation of socket contour was also observed with TAMP grafts (El Shazley et al., 2016). In addition, GlassBONE™ (Noraker, France), a synthetic resorbable bioactive glass 45S5 ceramic, has been successfully used for alveolar cleft reconstruction, with satisfactory healing found in two-thirds of tested patients (Graillon et al., 2018).

When bioactive glass is grafted, a significant increase in bone density is noted 6 months after the cessation of tooth movement; this finding may be attributed to the beneficial effects of alkalization on collagen synthesis and hydroxyapatite formation (Shoreibah et al., 2012). In addition, a marked reduction in orthodontic treatment duration was associated with bioactive glass grafting. Periodontal health was also enhanced with negligible apical root resorption and improved probing depth (Shoreibah et al., 2012). Although bioactive glass does not provide the same level of bone density as bovine-derived xenograft, both materials decrease the duration of orthodontic treatment and reduce the risk of root resorption (Bahammam, 2016).

Bioactive glass has also been applied with other grafting materials. For example, a case report from 2000 described how grafting a DFDBA-granular bioactive glass (1:1) mixture in the buccal aspect of the edentulous cleft region of a patient with cleft lip and palate resulted in good bone regeneration and successful orthodontic tooth movement into the grafted site (Yilmaz et al., 2000). However, these results should be interpreted with caution as they are derived from a single case report.

## Platelet-Rich Fibrin (PRF)

Endogenous biomaterials have been developed to overcome the limitations associated with current clinical approaches for autografting. PRF is a cost-effective material (Miron and Choukroun, 2017) that is increasingly being used for regenerative dentistry, specifically next-generation autologous platelet therapy (Liu et al., 2019). PRF contains stem cells, growth factors, and cytokines and is obtained through a minimally invasive procedure that centrifuges whole blood without additives (Choukroun et al., 2006). It can modulate inflammation and enhance the healing process, thereby promoting the regenerative capacity of the periosteum (Miron and Choukroun, 2017). In addition, its dense, protein-rich fibrin mesh functions as a three-dimensional fibrous scaffold for cell migration and a retainer for sustained growth factor release (Karimi and Rockwell, 2019).

Both animal (Sar et al., 2019) and clinical studies (Tehranchi et al., 2018) show that PRF significantly accelerates alveolar bone turnover and orthodontic tooth movement, especially at the beginning of orthodontic treatment (Tehranchi et al., 2018). However, 15% of grafted patients experience severe pain attributable to PRF application (Tehranchi et al., 2018), highlighting the need for further investigation. Although the clinical applications of PRF in regenerative dentistry have grown in recent years (Miron and Choukroun, 2017), its application in orthodontics is limited. It is largely unknown if the content variation of PRF from different patients or the same patient at different health statuses will impact its outcome as a graft in orthodontic treatment. Additionally, since PRF contains donor cells, it is not suitable to be used as an allograft. Its usage as an autograft material is also limited by availability when extracted from the patient’s blood (Choukroun et al., 2006).

## Pluri and Multipotent Cells

Over the last few decades, multiple pluri- and multi-potent cells have been explored for use in bone augmentation (Li C. et al., 2021; Holly et al., 2021). Bone marrow is the main source of MSCs for clinical applications; in fact, bone marrow-derived mesenchymal stromal cells (BM-MSCs) were the first MSCs to be discovered (Strioga et al., 2012). Compared to iliac crest bone grafts, resorbable collagen sponges combined with BM-MSCs provide similar bone healing results in the closure of alveolar cleft defects with reduced donor site morbidity and decreased donor site pain intensity and frequency (Gimbel et al., 2007).

Recently, successful bone regeneration has been reported using autogenous BM-MSCs in a dog model of an artificial alveolar cleft. In this study, new bone formation was achieved, thereby allowing orthodontic tooth movement beyond the anatomical limit (Tanimoto et al., 2015). Furthermore, a consistent rate of orthodontic tooth movement was observed in the experimental group compared to varied rates in the control group (Tanimoto et al., 2015), suggesting that MSCs in bone graft materials may have a modulatory effect on the bone remodeling process during orthodontic treatment. In alignment with this observation, the expression of RANKL, a molecule that regulates osteoclastic differentiation, was significantly increased in BM-MSCs under compressive stress (Wang et al., 2021). This finding suggests that BM-MSCs may accelerate tooth movement by expressing cytokines that promote osteoclastogenesis.

Due to ease of accessibility, dental-derived MSCs have gained attention in the past few years and have entered clinical trials (Paz et al., 2018). First isolated from the dental pulp of extracted third molars, dental-derived MSCs have now been purified from various dental tissues, including pulp tissue of permanent teeth and exfoliated deciduous teeth, apical papilla, periodontal ligament, gingiva, dental follicle, tooth germ, and alveolar bone (Gan et al., 2020). Dental-derived MSCs not only display the same characteristics as BM-MSCs but also possess immunomodulatory and anti-inflammatory advantages in the local dental tissue environment (Spagnuolo et al., 2018). Tanikawa *et al.* utilized autologous deciduous dental pulp stem cells for maxillary alveolar reconstruction and achieved progressive alveolar bone union without grafting site complications in cleft lip and palate patients (Tanikawa et al., 2020). Previous studies have also suggested that gingival-derived MSCs have great potential for repairing alveolar bone defects (Gao and Cao, 2020; Kandalam et al., 2021). However, the impact of dental-derived MSCs on orthodontic tooth movement is not yet well understood.

## Materials With 3D Printed Scaffolds

Conventional bone grafts, such as allografts and xenografts, often fail to provide the support necessary to maintain the desired generated tissue volume, especially under the mechanical forces in the oral cavity (Seciu et al., 2019). This is particularly challenging for vertical bone augmentation or personalized esthetic bone reconstruction, where highly tailored bone contours and structural stability are required. To overcome this obstacle, materials with three-dimensional architecture mimicking the anatomical and histological arrangement of natural bone have been developed (Kim et al., 2010).

Recent advances in microfabrication, particularly 3D bio-printing, support the construction of complex structures from bioactive/biodegradable materials, including polymers, bioceramics, and composites [as reviewed in (Asa’ad et al., 2016)]. In a recent study, a 3D-printed calcium phosphate scaffold was fabricated according to the geometry of artificial alveolar clefts in rats and showed promising scaffolding and osteoconductive properties (Korn et al., 2020). A 3D-printed custom hydroxyapatite/TCP graft supplied with rhBMP2 also achieved bone regeneration to the same level of rhBMP2-coupled deproteinized bovine bone material (Bio-Oss^®^) (Ryu et al., 2021). Although the exact mechanism of how 3D-printed scaffolds benefit orthodontic tooth movement remains unmapped, evidence suggests that grafting with 3D-printed scaffolds may offer enhanced orthodontic outcomes.

## Conclusion and Future Directions

Optimizing esthetics, providing functional and comfortable occlusion, and improving overall health are all goals of successful orthodontic treatment, for which preservation of the alveolar bone is a crucial limiting factor. Most materials reviewed in this article mediate accelerated orthodontic tooth movement and thus can reduce treatment duration and cost. These features are particularly attractive to patients facing extended treatment times, such as those in need of tooth extractions and additional periodontal support. Although a quantitative report is not currently realistic due to the limited available research to date, qualitatively analyzing pre-clinical novel materials will provide insight for their future usage in regenerative orthodontics. High-quality randomized controlled trials with larger sample sizes and longer follow-up periods are nevertheless warranted for translating these novel biological concepts into clinical practice. In our opinion, future exploration should also aim to reveal the potential long-term complications of these materials, as well as their impacts on growth and development in adolescents.

A rising number of reports suggest that adjunct treatments can support grafting and have the potential to improve orthodontic treatment. For example, the possibility of vibration accelerating orthodontic tooth movement has been a hot study topic over the last decade (Telatar and Gungor, 2021; Mayama et al., 2022). At the same time, studies have shown that high-frequency vibration treatment increases osteogenic differentiation of human BM-MSCs *in vitro* (Pre et al., 2013) and low-level mechanical vibration stimulates osteogenesis and osteointegration of porous titanium implants in the repair of long bone defects (Jing et al., 2015). In addition, low-intensity pulsed ultrasound (LIPUS) has been proven to accelerate new alveolar bone formation in a periodontal injury animal model (Wang et al., 2018) and enhance BM-MSCs-based periodontal regenerative therapies (Wang et al., 2022). Moreover, LIPUS can shorten the overall duration of orthodontic treatment (Kaur and El-Bialy, 2020) and minimize orthodontically-induced tooth root resorption (El-Bialy et al., 2020). Last but not least, laser photobiomodulation in combination with PRF demonstrated better bone healing than PRF alone in an iliac crest critical-sized bone defect sheep model (Surmeli Baran et al., 2021). Photobiomodulation was also found to enhance bone formation of hydroxyapatite biomaterial in the dental alveolus in an experimental extraction rat model (Dalapria et al., 2022). On the other hand, the effects of photobiomodulation on orthodontic treatment have started to attract attention (Li J. et al., 2021; Yavagal et al., 2021). In all, a detailed assessment of the influence of adjunct treatments with different grafting materials on orthodontic tooth movement is warranted to further optimize treatment outcomes.
